# Population-based statistical inference for temporal sequence of somatic mutations in cancer genomes

**DOI:** 10.1186/s12920-018-0352-z

**Published:** 2018-04-20

**Authors:** Je-Keun Rhee, Tae-Min Kim

**Affiliations:** 1 Cancer Research Institute, College of Medicine, The Catholic University of Korea, 222 Banpo-daero, Seocho-gu, Seoul, 06591 Republic of Korea; 2 Department of Medical Informatics, College of Medicine, The Catholic University of Korea, 222 Banpo-daero, Seocho-gu, Seoul, 06591 Republic of Korea

**Keywords:** Somatic mutation, Cancer genome, Mutation accumulation

## Abstract

**Background:**

It is well recognized that accumulation of somatic mutations in cancer genomes plays a role in carcinogenesis; however, the temporal sequence and evolutionary relationship of somatic mutations remain largely unknown.

**Methods:**

In this study, we built a population-based statistical framework to infer the temporal sequence of acquisition of somatic mutations. Using the model, we analyzed the mutation profiles of 1954 tumor specimens across eight tumor types.

**Results:**

As a result, we identified tumor type-specific directed networks composed of 2-15 cancer-related genes (nodes) and their mutational orders (edges). The most common ancestors identified in pairwise comparison of somatic mutations were *TP53* mutations in breast, head/neck, and lung cancers. The known relationship of *KRAS* to *TP53* mutations in colorectal cancers was identified, as well as potential ancestors of *TP53* mutation such as *NOTCH1*, *EGFR*, and *PTEN* mutations in head/neck, lung and endometrial cancers, respectively. We also identified apoptosis-related genes enriched with ancestor mutations in lung cancers and a relationship between *APC* hotspot mutations and *TP53* mutations in colorectal cancers.

**Conclusion:**

While evolutionary analysis of cancers has focused on clonal versus subclonal mutations identified in individual genomes, our analysis aims to further discriminate ancestor versus descendant mutations in population-scale mutation profiles that may help select cancer drivers with clinical relevance.

**Electronic supplementary material:**

The online version of this article (10.1186/s12920-018-0352-z) contains supplementary material, which is available to authorized users.

## Background

Various types of genomic aberrations accumulate in cancer genomes and play roles in the development and progression of the disease [1]. It has long been recognized that cancer genomes undergo a stepwise progression in which they acquire somatic mutations in a sequential order during their evolution. This model is relatively well established in colorectal cancer genomes [2], and may be true for other types of cancer. Recent advances in high-throughput sequencing technologies have enabled screening of cancer genomes for well-known cancer-related genomic aberrations such as somatic mutations, DNA copy number alterations, and chromosomal translocations [3]. Genomic snapshots of human solid tumors can only be obtained by surgical intervention and such procedures have limitations for a full understanding of the temporal or longitudinal evolution of individual cancer genomes. While current cancer genome studies are mainly focused on the identification of significant recurrent genomic aberrations as potential cancer drivers, the inference of acquisition order of somatic mutations may provide mechanistic insights into the evolution of the cancer genome and have potential clinical relevance.

Several studies have been proposed to investigate the order of acquisition of genomic alterations. For example, Attolini et al. proposed a mathematical approach to determine the sequential order of *APC*, *KRAS*, and *TP53* mutations in 70 colorectal cancer samples [4]. They estimated the mutation rate per allele and predicted the temporal sequences for mutations acquired in these genes. Hu et al. tried to identify tumor driver genes using association rule mining [5]. In addition, some researchers tried to estimate the tumor initiation time. Tomasetti et al. modeled the process of mutation accumulation and verified that a substantial number of somatic mutations may have appeared before the onset of neoplasia [6]. Also, Foo et al. investigated driver mutations in the evolutionary processes of mutation accumulation using healthy and tumor tissues [7]. However, most of these previous reports used binary genomic data (e.g., calls for presence or absence of mutations or copy number alterations) and did not exploit information regarding the clonality of mutations (e.g., clonal vs. subclonal mutations).

Here, we inferred the acquisition order of somatic mutations (hereafter in this study, we define somatic mutations as non-silent single nucleotide variations [SNVs] including missense, nonsense, and splice site mutations) based on information of the cancer cell fraction (CCF) measured for each mutation, using 1954 tumor specimens from eight major tumor types of the Cancer Genome Atlas (TCGA) consortium: 90 samples from bladder urothelial carcinoma (BLCA); 733 from breast invasive carcinoma (BRCA); 246 from colorectal adenocarcinoma (COADREAD); 265 from head and neck squamous cell carcinoma (HNSC); 42 from kidney renal clear cell carcinoma (KIRC); 290 from lung adenocarcinoma (KIRC); 118 from lung squamous cell carcinoma (LUSC); and 170 from uterine corpus endometrial carcinoma (UCEC). The CCFs, as variant allele frequencies (VAFs) adjusted for the tumor purity and global/local ploidy, are a measure of the clonality of given somatic mutations. CCFs have been used to distinguish clonal or subclonal mutations in individual cancer genomes [8]. In theory, clonal mutations represent early genomic events that have occurred in a founder cell and are maintained during the clonal proliferation whereas subclonal mutations represent late genomic events that are not yet fixed by clonal amplification or clonal sweeps. Under the infinite sites model of genome evolution with no homoplasy, somatic mutations with lower CCFs cannot occur earlier than those with higher CCFs [9]. Although a recent study showed that the mutation acquisition order affects cancer and cancer therapy [10], it is still largely unclear how to aggregate the information on individual genomes such as the CCFs to facilitate population-scale inference of temporal ordering of somatic mutations. In this study, we established a statistical model to infer the temporal order of somatic mutations observed across multiple cancer genomes and applied the method to a pan-cancer landscape of somatic mutations of eight major tumor types.

## Methods

### Study dataset

All experiments were carried out using publicly available TCGA pan-cancer data for eight tumor types, BLCA, BRCA, COADREAD, HNSC, KIRC, LUAD, LUSC, and UCEC. All mutation data were obtained from mutation annotation format (MAF) files with available sequencing read abundance of mutant and wildtype alleles to calculate VAFs. Among these somatic mutations, only the non-silent mutations (nonsense, mutation, and splice site SNVs) were extracted. We further selected mutations with minimum number of variant alleles ≥ 5 and minimum number of total alleles ≥ 30. The integer-level copy number, tumor ploidy and purity values estimated by ABSOLUTE [11] were downloaded from the Synapse website for TCGA pancancer analysis (https://www.synapse.org/\#!Synapse:syn1703335) and used for the estimation of CCFs.

### Estimating cancer cell fraction

The CCF is defined as the proportion of cancer cells harboring the mutations for each variant, and can be estimated using a method outlined by Landau et al. [12]. Briefly, for a single point mutation *m*_*i*_ at a sample *n*, $P\left (C^{n}_{i}\right)$, the posterior distribution for the CCF $C^{n}_{i}$ is obtained from binomial distribution of the observed VAF over the expected VAFs calculated using a uniform grid of 100 CCF values ($C^{n}_{i} \in $ [0.01, 1]), and subsequently normalized. Then, the probability mass function of the $P\left (C^{n}_{i}\right)$ is represented as:

1 $$ P\left(C^{n}_{i}\right) = \sum\limits^{1}_{k=0.01} P\left(C^{n}_{i}=k\right)  $$

### Statistical inference of mutational temporal order of somatic mutations

The mutational order for a pair of somatic mutation, *m*_*i*_ and *m*_*j*_, was determined using a generalized likelihood ratio test (GLRT). This examines whether the occurrence of somatic mutation *m*_*i*_ precedes that of another somatic mutation *m*_*j*_. Then, a null hypothesis *H*_0_ and an alternative hypothesis *H*_1_ follow:

$$H_{0}: m_{i}~\text{is an ancestor of}~ m_{j} (m_{i} \to m_{j}) $$

$$H_{1}: m_{i}~\text{is not an ancestor of}~ m_{j} $$

Suppose that there are a total of *N* samples, which have somatic mutations both in *m*_*i*_ and *m*_*j*_. The CCF for *m*_*i*_ and *m*_*j*_ is represented as a set of independent and identically distributed (i.i.d.) variables, as $C_{i}=\left ({C}^{1}_{i},\ C^{2}_{i},\ \dots,\ C^{N}_{i}\right)$ and $C_{j}=\left ({C}^{1}_{j},\ C^{2}_{j},\ \dots,\ C^{N}_{j}\right)$, respectively. In the *n*-th sample among the total *N*, the evolutionary precedence of the two mutations, *m*_*i*_ and *m*_*j*_, was approximated from the comparison of their CCFs, $C^{n}_{i}$ and $C^{n}_{j}$, respectively. That is, $C^{n}_{i} \geq C^{n}_{j}$ implies that mutation *m *_*i*_ is an ancestor of the mutation *m*_*j*_ in the sample *n*. Suppose that a random variable $D^{n}_{ij}$ represents the difference of two variables, $C^{n}_{i}$ and $C^{n}_{j}$, and $\hat {C^{n}_{i}}=P\left (C^{n}_{i}\right)$ and $\hat {C^{n}_{j}}=P\left (C^{n}_{j}\right)$ are the estimated distribution of the CCFs at a mutation *m*_*i*_ and *m*_*j*_, respectively, in the sample *n*. Then the two hypotheses can be re-written as follows:

$$\mathrm{H}_{0}: \hat{D^{n}_{ij}}\geq\thinspace0 $$

$$\mathrm{H}_{1}: \hat{D^{n}_{ij}}<\thinspace0 \,, $$

where $\hat {D^{n}_{ij}}\ $is the estimated distribution of *D*_*ij*_ at the sample *n*. By the definition of GLRT under i.i.d. condition, the statistics are represented as:

2 $$\begin{array}{@{}rcl@{}} \Lambda &=& \frac{max_{H_{0}}L\left(\hat{D^{1}_{ij}}, \hat{D^{2}_{ij}},..., \hat{D^{N}_{ij}}\right) }{max_{{H_{0}}\cup{H_{1}}}L\left(\hat{D^{1}_{ij}}, \hat{D^{2}_{ij}},..., \hat{D^{N}_ {ij}}\right)} \\ &=& \prod\limits_{n=1}^{N} \frac{max_{H_{0}}L\left(\hat{D^{n}_{ij}} | {D^{n}_{ij}} \in [0,+\infty]\right)}{max_{{H_{0}}\cup{H_{1}}}L\left(\hat{D^{n}_{ij}} | {D^{n}_{ij}} \in [-\infty,+\infty]\right)}\,, \end{array} $$

where *L*(·) is a likelihood function. Using the characteristics of the convolution of two independent random variables, the probability mass function of the variable $D^{n}_{ij} \left (D^{n}_{ij} = C^{n}_{i} - C^{n}_{j}\right)$ is expressed as

3 $$ P(D^{n}_{ij}=z)=\sum\limits^{1}_{k=0}{P\left(C^{n}_{i}=k\right)P\left(C^{n}_{j}=-z+k\right)}  $$

Then, Eq. 2 is rewritten with the property of Eq. 3 as follows:

4 $$ \Lambda = \prod\limits_{n=1}^{N} \frac{max_{H_{0}}\sum_{z=0}^{+\infty}P\left(\hat{D^{n}_{ij}}=z)\right)}{max_{{H_{0}}\cup{H_{1}}}\sum_{-\infty}^{+\infty}P\left(\hat{D^{n}_{ij}}=z)\right)}  $$

Equation 4 with k ∈ [0.01, 1] is rewritten as

5 $$ \Lambda = \prod\limits_{n=1}^{N} \frac{max_{H_{0}}\sum_{z=0}^{1}P\left(\hat{D^{n}_{ij}}=z)\right)}{max_{{H_{0}}\cup{H_{1}}}\sum_{-1}^{1}P\left(\hat{D^{n}_{ij}}=z)\right)}   $$

For the convenience of the calculation, the statistics of Eq. 5 are changed to a logarithmic value:

6 $$\begin{array}{@{}rcl@{}} log(\Lambda)_{I_{D_{max}<0}} \!\!&\!=&\!\!\sum\limits_{n=1}^{N}log\left(\frac{max_{H_{0}}\sum_{z=0}^{1}P\left(\hat{D^{n}_{ij}}=1\right)}{max_{{H_{0}}\cup{H_{1}}}\sum_{z=-1}^{1}P\left(\hat{D^{n}_{ij}}=1\right)}\right){I_{\hat{{D_{ij}^{n}}_{max}}<0}} \\ \!\!&=& \!\!\sum\limits_{n=1}^{N}\left(log\left(max_{H_{0}}\sum_{z=0}^{1}P\left(\hat{D^{n}_{ij}}=1\right)\right)\right. \\ \!\!&& \left. \! \!- log\left(max_{{H_{0}}\cup{H_{1}}}\!\!\sum_{z=-1}^{1}\!\!P\left(\hat{D^{n}_{ij}}\,=\,1\right)\right)\right){I_{\hat{{D_{ij}^{n}}_{max}}<0}} \end{array} $$

$I_{{{\hat {D^{n}_{ij}}}_{max}}<0}$ is an indicator function for whether the maximum value of the likelihood function for $\hat {D^{n}_{ij}}$ is observed at *z* < 0. $I_{{{\hat {D^{n}_{ij}}}_{max}}<0} = 0$ means that the numerator and denominator values are identical. The null hypothesis(*H*_0_) is not rejected if the log(*Λ*) is significantly large.

The statistical tests were carried out using mutant gene pairs with number of cases > 10. The statistical cutoff for the log(*Λ*) was obtained by random re-arrangement of the original data to generate a background distribution of the GLRT statistics. The cutoff was determined as a 5th percentile value from the background distribution of the 100,000 randomized experiments.

## Results

### Co-occurring pairs of somatic mutations

**Fig. 1:**
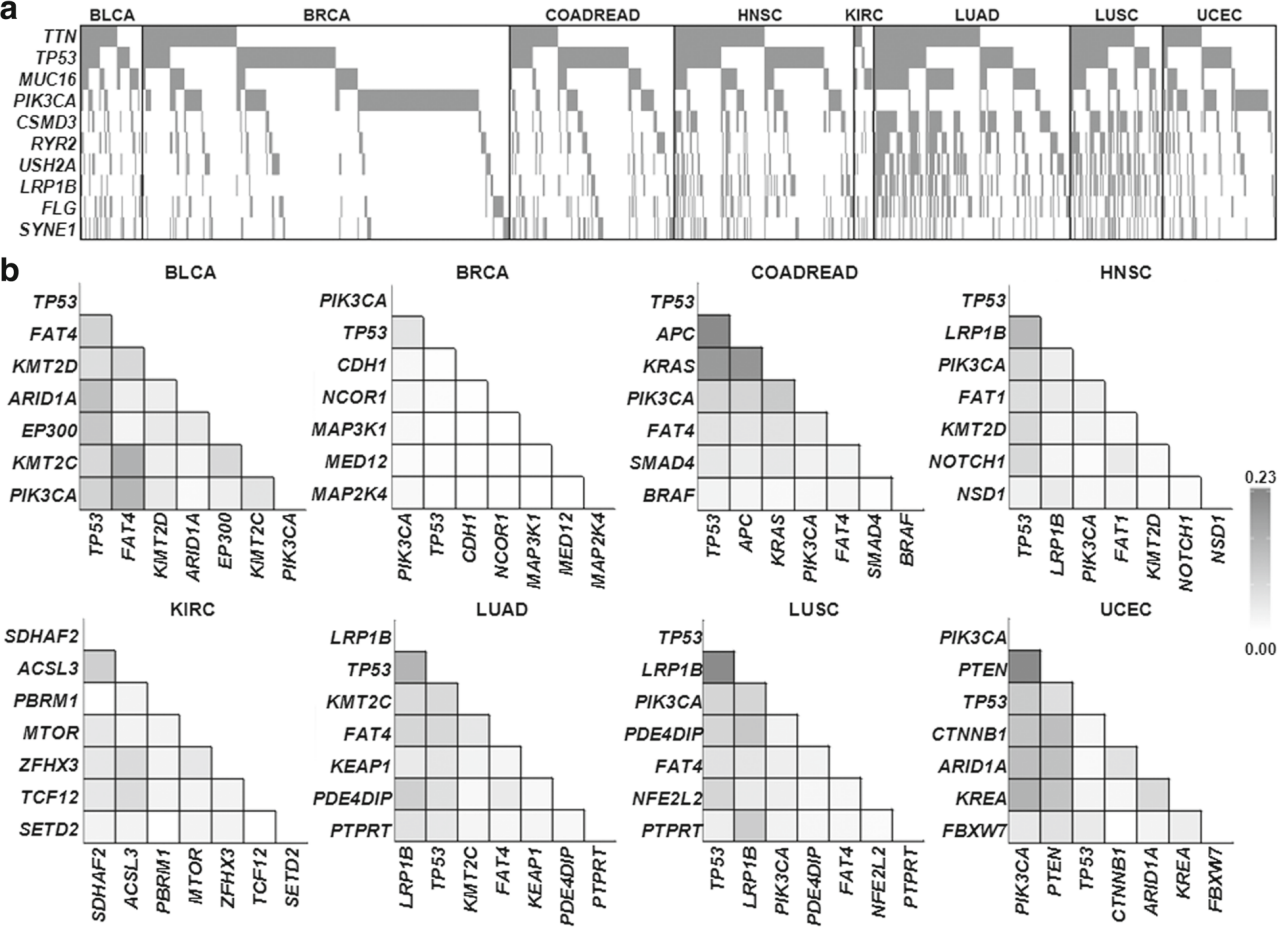
Frequent somatic mutations and co-occurring mutation pairs. **a** Distribution of somatic mutations observed in all cases examined. The plot is shown for the top 10 frequently mutated genes across all cases. A tumor patient with a somatic mutation is marked as gray in each column. **b** Frequently observed gene pairs of somatic mutations are shown for each tumor type. The heatmaps of SCORE-CO are shown for the top 7 genes among CGC in a given tumor type. The color indicator of SCORE-CO is shown separately (right)

We first examined genes with frequent mutations and their co-occurrence patterns using mutation profiles of 1954 patients across eight TCGA tumor types (BLCA, BRCA, COADREAD, HNSC, KIRC, LUAD, LUSC, and UCEC). A mean number of 120 somatic mutations (nonsilent SNVs; missense, nonsense and splice site mutations) were observed (1 to 1597 mutations per case; median of 59 mutations; Table 1). Figure 1(a) shows the distribution of somatic mutations for 10 genes with the most frequent somatic mutations across all cases examined. To investigate gene pairs, we employed a scoring system of mutation co-occurrence. The score of the co-occurrence, SCORE-CO is calculated by summing the outputs of the logical conjunction (’AND’ gate) for the binary input data as the presence or absence of the somatic mutations in the given pair of two genes and by dividing the value by the number of total cases in the dataset. Table S1 shows the co-occurring pairs of somatic mutations observed in no fewer than 10 cases per tumor type (Additional file 1: Table S1). The gene pairs with high SCORE-CO include *TTN* and *MUC16* whose frequent mutations are largely due to their large gene size (36,800 and 14,500 amino acids, respectively) rather than their functional significance. Thus, we focused on mutations in known cancer-related genes or the Cancer Gene Census (CGC) [13] (Fig. 1(b)). The co-occurring mutation gene pairs with high SCORE-CO were tumor type-specific, e.g., gene pairs of *TP53* and *PIK3CA* were highly ranked in BLCA, BRCA, COADREAD, HNSC, LUSC, UCEC (SCORE-CO = 0.089 for 8 cases with the co-occurrence / total 90 patients, 0.055 for 40 cases, 0.085 for 21 cases, 0.083 for 22 cases, 0.085 for 10 cases, 0.106 for 18 cases, respectively) and to a lesser extent in LUAD (SCORE-CO =0.024 for 7 cases). However, the pair of *TP53* and *PIK3CA* was not identified in KIRC. *LRP1B* mutants frequently co-occurred with *TP53* mutants in HNSC, LUAD and LUSC. *APC* mutants were frequently observed with *TP53* or *KRAS* mutations in COADREAD. In addition, some of the mutation occurrences were tumor type-specific, e.g., *PIK3CA* mutations showed co-occurrence with *FAT4* mutations with a high frequency in BLCA, but mainly co-occurred with *PTEN* mutations in UCEC.

**Table 1:** Number of non-silent mutations in each tumor type

	Nonsense	Missense	Splice site
	mutation	mutation	mutation
BLCA	1430	14,685	560
BRCA	2512	30,499	815
COADREAD	2348	37,702	765
HNSC	2110	26,040	1179
KIRC	185	2592	513
LUAD	4310	53,898	3090
LUSC	2004	23,485	640
UCEC	1790	21,327	538

### Temporal sequence of mutations in cancer-related genes

To infer the temporal sequence of the somatic mutations in cancer-related genes, we first distinguished two types of mutations, clonal and subclonal mutations, based on CCF. The distinction was made by a criterion proposed by Landau et al. [12], and the measure of CCF was clearly higher for the clonal mutations than for subclonal mutations (*P* value <1.0∗10^−20^).

**Fig. 2:**
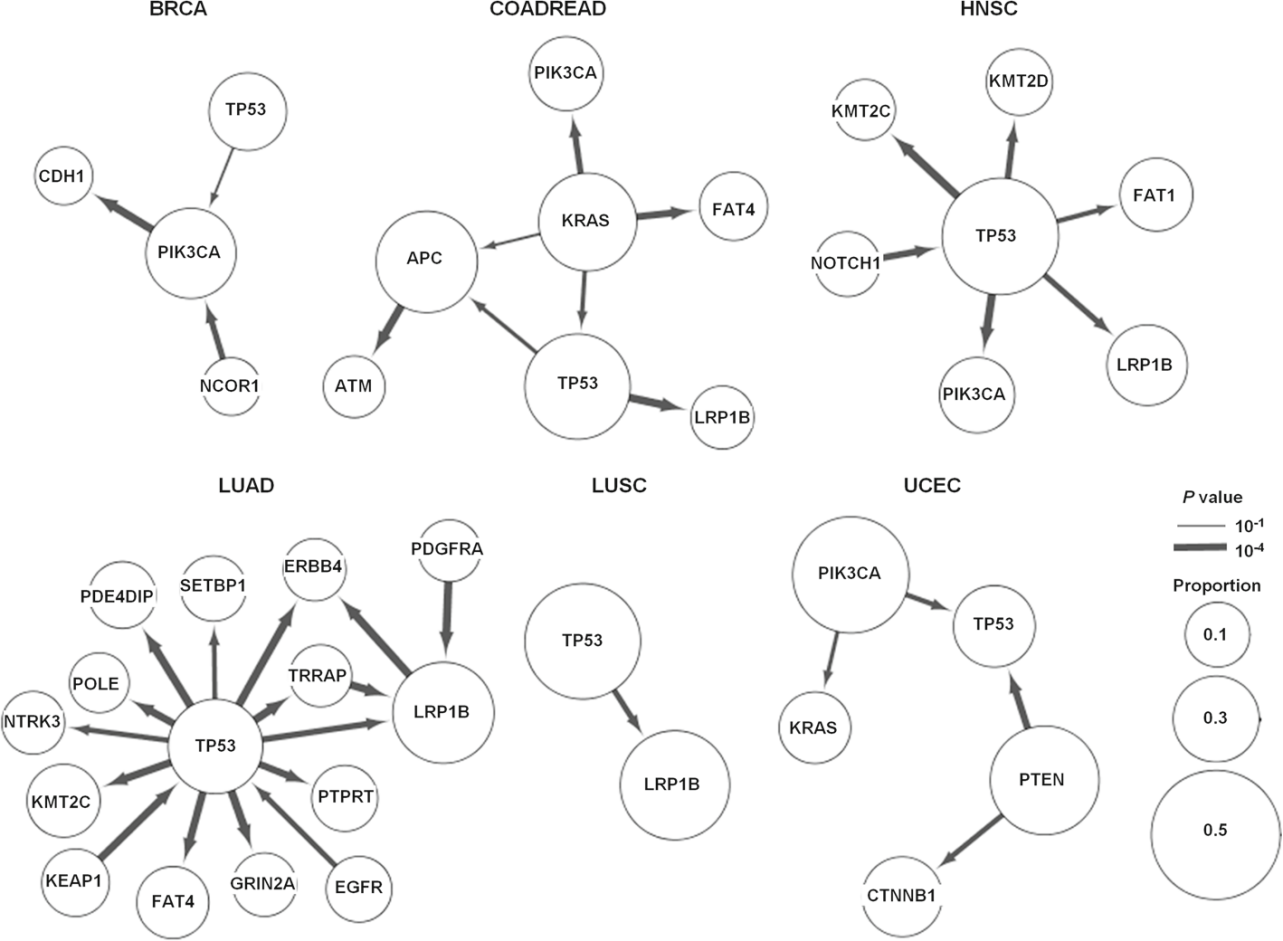
Tumor type-specific networks representing the mutation order among cancer-related genes. The thickness of the edges is proportional to the −*logP* value and the size of the nodes corresponds to the proportion of the genes with somatic mutations

Based on the CCF, we established a statistical framework for the population-based inference of temporal order between somatic mutations in a gene pair and applied the method for the mutation profiles from individual tumor types. Using the results from permutation tests to determine the minimum case number to identify the statistically significant mutation pairs with sequential orders (i.e., *log*(*Λ*)≈ 0 at the 5th percentile of the 100,000 re-sampling experiments at the small number of cases), we performed the test for all pairs of somatic mutations observed in no fewer than 10 cases (Additional file 2: Figure S1 (a)). Additional file 2: Figure S1 (b) shows the distribution of the number of the mutation pairs in each tumor type. The ancestor-descendant relationship in a mutation pair was then inferred by GLRT statistics and the significance was estimated for each direction. Figure 2 shows the mutation orders of cancer-related genes for six tumor types except for BLCA and KIRC, which did not have any significant mutant pairs within cancer-related genes (*P* value cutoff was 0.05).

The mutation order of gene pairs was largely distinct across tumor types, suggesting that the accumulation patterns or hierarchy of somatic mutations are lineage-dependent. However, some of the mutation pairs and their orders were consistently observed across tumor types. For example, a frequently co-occurred mutation pair of *TP53* and *LRP1B* (Fig. 1(b)) was observed as ancestor (*TP53*) - descendant (*LRP1B*) pairs (*TP53* →*LRP1B*) in COADREAD, HNSC, LUAD, and LUSC, with statistical significance (*P* value =3.0∗10^−5^, 0.00044, 0.00029 and 0.00058, respectively). In addition, *KMT2C* mutation was consistently observed as a descendant of *TP53* mutations (*TP53* →*KMT2C*) in HNSC and LUAD (*P* value =3.0∗10^−5^ and 5.0∗10^−5^, respectively).

When we investigated mutation pairs in each tumor type, three ordered pairs were identified with statistical significance in BRCA (*P* value < 0.05) (Fig. 2). The hierarchy of the three genes (*TP53* →*PIK3CA* →*CDH1*) in BRCA suggests that *TP53* mutations represent early events that are followed by subsequent *PIK3CA* mutations (*P* value =0.034), then *CDH1* mutations (*P* value =3.0∗10^−5^). This mutation sequence can be functionally interpreted as follows: genomic integrity is disrupted with *TP53* mutations followed by cancer cell proliferation stimulated by *PIK3CA* mutations and the acquisition of later invasive/metastatic potential with *CDH1* mutations. This mutation order between *TP53* and *PIK3CA* was also found in HNSC (*P* value =3.0∗10^−5^), suggesting that the *TP53* →*PIK3CA* axis may play important roles in the development of epithelial tumors. *NCOR1* mutation was also observed as an ancestor for *PIK3CA* mutation (*P* value =0.00036) and it has been reported that functional inactivation of *NCOR1* as a *HDAC3* cofactor may produce genomic instability, which is functionally equivalent to the loss of *TP53* [14].

For LUAD, 16 ordered pairs were identified with statistical significance. The elevated mutation abundance (mean of 211 mutations per LUAD case vs. mean of 120 mutations for total cases) and the relatively large size of the cohort (290 cases) may explain this number, but only one mutation pair was observed in LUSC with similar mutation abundance (average 221 mutations per cases) and a smaller number of cohorts (118 cases). *TP53* mutation appeared as a hub in the 16 edge-based network of LUAD and was identified as ancestor in most mutation pairs. *TP53* mutations have been implicated in tumor development and progression across many tumor types [15–17]. Our analysis also suggests that *EGFR* mutations may be earlier genomic events among the mutations in the LUAD pathogenesis [18]. A substantial fraction of *EGFR* mutations in LUAD are considered to be early addicted targets of targeted therapy [19, 20], suggesting that they represent early genomic aberrations together with *TP53* mutations. In the case of LUSC, the *TP53* →*LRP1B* ordered mutation pair was solely observed.

In HNSC, *NOTCH1* mutations may be earlier events than *TP53* mutations. Although it is mutated at a lower frequency than *TP53* in HNSC, *NOTCH1* has been highlighted as a potential cancer driver and tumor suppressor in HNSC [21].

For COADREAD, ordered pairs of somatic mutations involving *APC*, *KRAS* and *TP53* are observed and have been recognized to have pivotal roles associated with colorectal carcinogenesis [2]. Colorectal carcinogenesis is one of the well-established stepwise cancer progression models and involves sequential acquisition of *APC*, *KRAS*, and *TP53* mutations at colorectal dysplasia, adenoma, and carcinoma stages, respectively [22, 23]. Our inferred hierarchy from somatic mutations suggested that *KRAS* mutations were the earliest events in colorectal carcinogenesis. Given that our statistical model only considered the SNV, tumor suppressors that can be inactivated by chromosomal deletions, such as *APC*, may not be adequately assessed for order of mutation.

### Accumulation of somatic mutations including non-CGC gene

**Fig. 3:**
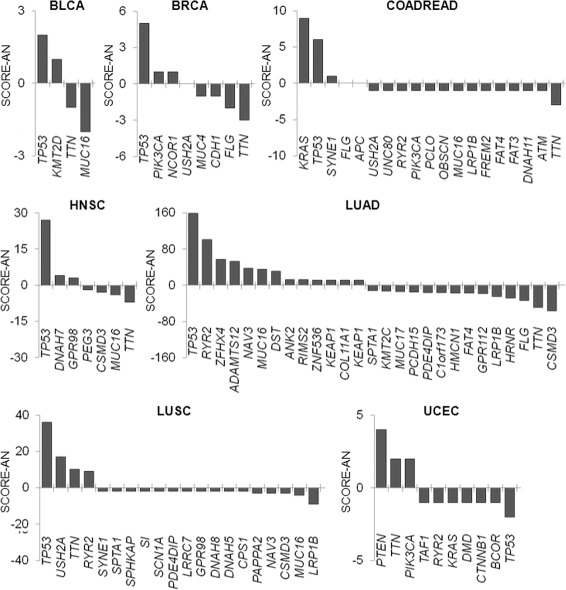
Ancestral scores of genes in each tumor type. The figure shows SCORE-AN (y-axis), calculated as the number of the ancestors minus the number of descendants for each gene. Genes are sorted in descending order of the SCORE-AN for each tumor type (x-axis). For LUAD, genes with absolute number of SCORE-AN > 10 are plotted, and for LUSC genes with absolute number of SCORE-AN > 2 are shown

We next performed analysis beyond the known cancer-related genes (Additional file 3: Table S2). For individual genes, we calculated SCORE-AN by subtracting the number of genes marked as a descendant from the number of genes marked as an ancestor in a given tumor type. SCORE-AN for the genes is provided in Additional file 4: Table S3 and Fig. 3. A large positive value of the SCORE-AN means that the corresponding gene is more likely to be an ancestor or early clonal event and can be regarded as a potential driver of the corresponding tumor type. In contrast, the genes with a large negative value would be passengers or late mutation events.

Consistent with our assumption, many of the genes with high SCORE-AN values or genes with more calls for ancestors were cancer-related genes listed in CGC (Fig. 3). For example, the genes with a positive SCORE-AN were *TP53* and *KMT2D* in BLCA and *TP53*, *PIK3CA* and *NCOR1* in BRCA. However, the two longest genes in the human genome, *TTN* and *MUC16*, which are likely to be passengers without putative roles generally showed negative values of SCORE-AN even though these two genes showed frequent mutations as shown in Fig. 1(a). In the case of UCEC, *TTN* and *TP53* showed a positive and negative value, respectively. For this tumor type, *TP53* mutation was marked as a descendant for *PIK3CA* and *PTEN* mutations. It was also noted that *TP53* mutations were also observed as descendants of *KRAS* mutations in COADREAD. For theses tumor types (UCEC and COADREAD), an unusual tendency for elevated mutation rates was observed and this might be responsible for the unique evolutionary position of *TP53* [24].

To further evaluate the potential functional significance of the SCORE-AN, we carried out Gene Set Enrichment Analysis (GSEA) [25] to identify the functional gene sets significantly enriched for genes with high or low SCORE-AN. Additional file 5: Table S4, Additional file 6: Table S5, Additional file 7: Table S6, and Additional file 8: Table S7 show the GSEA results only for LUAD, in which there was a high enough number of the ranked genes (427 genes) for analysis. The results for positively ranked genes on C5bp predefined gene sets (gene ontology, biological process in MSigDB), showed that most of the enriched gene sets were related to cell cycle and cellular transport (Additional file 5: Table S4). For gene sets enriched with low SCORE-AN, the significantly enriched genes were related to epidermal or epithelial development (Additional file 6: Table S5). On C2cp gene sets (canonical pathway in MSigDB), the results for the positively ranked genes or functions enriched with high SCORE-AN were also obviously enriched for cancer-related functionalities (Additional file 7: Table S6) but no gene sets with statistical significance were observed for genes with low SCORE-AN (Additional file 8: Table S7).

### Mutational hotspots and accumulation order

We next investigated somatic mutations occurring on known mutation hotspots and their temporal mutation order. Chang et al. previously defined 470 mutational hotspots in 275 genes and we investigated all of the pairs of hotspot mutations to detect the temporal sequence of mutations on known hotspots [26]. However, the number of mutation pairs harboring the hotspot mutations was too small for our GLRT-based statistical models (number of cases ≤ 5).

It has been previously reported that *APC* mutations may initiate the process of colon cancer development as one of the earliest genomic aberrations [27], but we did not obtain clear results for the early occurrence of *APC* mutations in the gene-level experiments shown in the previous section. We divided the *APC* mutations into hotspot mutations and non-hotspot mutations and then investigated the CCF distribution. The *APC* non-hotspot mutations showed relatively low CCF values compared with the *APC* hotspot mutations (Additional file 9: Figure S2(a)). When we further examined four cases harboring both *APC:Q1387* hotspot mutations and *TP53* mutations, the *APC:Q1387* hotspot mutations had higher CCF values compared with *TP53* mutation, and it is reasonable to assume that the *APC:Q1387* mutations would be an ancestor of the *TP53* mutations in these cases (Additional file 9: Figure S2(b)).

## Discussion

The identification of known and novel cancer drivers with moderate-to-high population-level frequency of somatic mutations has been one of the major goals in cancer genome analyses [28, 29]. However, the temporal sequence of somatic mutations, i.e., which somatic mutations occurred earlier in the evolution of cancer genomes than others, is still largely unknown. Early- and late-occurring somatic mutations have different biological and clinical implications-the early addicted somatic mutations may serve as appropriate targets for therapeutic intervention while late-occurring cancer drivers have been associated with therapeutic resistance or disease progression. The distinction of such early and late genomic events, especially for somatic mutations, has been previously investigated using VAF or CCF. However, VAF- or CCF-based discrimination of early/clonal and late/subclonal mutations is limited to an individual genome and may miss information inferring the temporal relationship between mutations. To solve this problem, we built a GLRT-based statistical framework to determine the temporal sequence of somatic mutations from mutation profiles of multiple individuals (population-level genomics data). This population-scale analysis may capture the temporal sequence of somatic mutations and identify the temporal sequence or hierarchy of somatic mutations of a given tumor type. Similar approaches have been previously proposed in which genomic data of multiple tumors (i.e., binary calls of chromosomal amplifications or deletions) at their fully transformed stages may be used to deduce the temporal sequence of genomic events (RESIC [4]); however, we extended this idea by exploiting the distinction of clonal versus subclonal mutations based on CCF estimates of individual mutations. We applied our method to publicly available mutation profiles of eight major human tumor types. For this, we carried out pairwise comparisons between somatic mutations based on a statistical test to infer the temporal order among them. Thus, it would be impossible to detect the effects of multiple factors on mutation acquisition. Although technical innovations have been proposed to solve this issue, e.g. single cell sequencing from a bulk tumor genome or longitudinal biopsies, these methods are largely limited in terms of cost or patient safety issues. Given that sequencing-based large-scale mutation profiles are currently available to the research community, such as those from the TCGA consortium used in our study, our method can be further applied to other datasets or tumor types. In addition, we assumed that *C*_*i*_ and *C*_*j*_ were under i.i.d. condition, and applied GLRT. Biologically, this assumption of independency between two mutations may not be valid given the crosstalk or interplay between genomic alterations in cancer cells. Other statistics for inference of mutational orders, such as an order statistic, can be considered as an alternative to GLRT.

Among the tumor types examined, we identified *TP53* mutations as a recurrently observed hub connected with other cancer-related genes, consistent with its prevalent and known roles in tumorigenesis across multiple cancer types [30]. Among the mutation pairs involving *TP53*, we observed the mutation pair of *KRAS* →*TP53* as a well-recognized mutation sequence in the stepwise colorectal carcinogenesis [2]. In addition, we recurrently observed the mutation pairs of *TP53* →*LRP1B* and *TP53* →*KMT2C* across multiple tumor types. Inactivation of *LRP1B* increased the invasive potential in an in vitro setting, implicating a role of *LRP1B* mutations in the later stages of carcinogenesis [31]. Whether the mutations in epigenetic modifiers are early or late events drivers is a subject of debate, with lines of evidence supporting early events for *TET2* mutations [32] or late events for *SETD2* mutations [33]. Our results suggest that *KMT2C* mutations are descendant genomic events relative to *TP53* mutations in LUAD and HNSC, but further experimental validation in terms of multiregion sequencing or other method is required. Moreover, as we expected, non-CGC genes were commonly observed as descendants of a CGC gene in multiple tumor types (Fig. 3 and Additional File 10: Table S8). For example, *USH2A* mutation was frequently observed in several tumor types as shown in Fig. 1a, but it was a late event occurring after *TP53* or *KRAS* mutation.

In the case of COADREAD, the mutation pair of *KRAS* →*APC* was observed even though it is generally recognized that *APC* mutations occur early, before *KRAS* and *TP53* mutations. One limitation of our methodology is that only SNVs available for CCF can be used as input of the algorithm. In the case of *APC*, chromosomal deletions or frameshifting indels may be also responsible for *APC* inactivation and our methods may not adequately evaluate the genetic hierarchy of tumor suppressors such as *APC*. When we limit the *APC* mutations to those on a known mutation hotspot (*APC:Q1387*) accompanying *TP53* mutations (four COADREAD cases), the CCF values of *APC* mutations were higher than those of *TP53* mutations suggesting that *APC* mutation may have occurred earlier than *TP53* mutation in those cases.

The population scale inference of mutational orders assumes that the mutation processes are uniform across the cases, or at least for the majority of cases. This assumption, and the related results, should be interpreted with caution since the sequence of mutation accumulation can be specific in individual cancer genomes and distinctive to patient subgroups, according to their tumor subtype or other clinical features. By collecting many more samples with information, the specific accumulation patterns (e.g., candidate sets of mutation orders) may be further investigated and might help elucidate individual features such as the treatment response.

## Conclusions

In spite of several limitations, our results inferred a genetic hierarchy between somatic mutations as part of the cancer genome evolution. We found some ordered pairs of genes within cancer-related genes in each tumor and this information will provide mechanistic insights into the tumor initiation process. We also demonstrated that the scores of mutation co-occurrence (SCORE-CO) or ancestor/descendant ratio (SCORE-AN) may help identify or prioritize new candidates of driver mutations in each tumor. Furthermore, the study on mutation hotspot information may be more robust in that the hotspot mutations represent functionally relevant cancer drivers as shown in the example of *APC* mutations in COADREAD. In summary, our proposed statistical framework can be used to infer the temporal sequence of somatic mutations in population-scale cancer genomics data, providing information regarding the timing of mutation occurrence in given tumor types.

## Additional files

Additional file 1 **Table S1.** SCORE-CO for the pair of genes in each tumor type. (XLSX 696 kb)

Additional file 2 **Figure S1.** Determination of the minimum sample size for the experiments (a) 5 percentile of the 100,000 random re-sampling experiments with the number of cases (b) Distribution of the frequency for the mutant gene pairs. (PDF 23 kb)

Additional file 3 **Table S2.** Ordered pairs with statistical significance in each tumor type. (XLSX 61 kb)

Additional file 4 **Table S3.** SCORE-AN for genes observed in each tumor type. (XLSX 22 kb)

Additional file 5 **Table S4.** Enrichment genesets on C5bp for genes with positive SCORE-AN. The results were obtained by GSEApreranked on C5bp (Gene Ontology, biological process). BLCA, BRCA and KIRC were no results with nominal *P* value <0.05. (XLSX 16 kb)

Additional file 6 **Table S5.** Enrichment genesets on C5bp for genes with negative SCORE-AN. The results were obtained by GSEApreranked on C5bp (Gene Ontology, biological process). BLCA, BRCA, COADREAD, HNSC, KIRC and LUSC were no results with nominal *P* value <0.05. (XLSX 10 kb)

Additional file 7 **Table S6.** Enrichment genesets on C2cp for genes with positive SCORE-AN. The results were obtained by GSEApreranked on C2cp (canonical pathway). BLCA, BRCA, COADREAD, HNSC and KIRC did not did not detect any genesets. (XLSX 12 kb)

Additional file 8 **Table S7.** Enrichment genesets on C2cp for genes with negative SCORE-AN. The results were obtained by GSEApreranked on C2cp (canonical pathway). BLCA, BRCA, COADREAD, KIRC and UCEC did not detect any genesets. (XLSX 11 kb)

Additional file 9 **Figure S2.** Hotspot mutation and CCF. (a) Maximum value of CCF in COADREAD. Each dot means a tumor patient with a somatic mutation in APC hotspots. (b) Distribution of CCFs for APC and TP53 mutations in COADREAD. The figure is plotted for the patients with APC:Q1387 hotspot mutation. Red is CCF distribution for APC and blue is for TP53. (PDF 35 kb)

Additional file 10 **Table S8.** Common temporal orders in multiple tumor types. (XLSX 11 kb)
