# Supplementary material for: Population-based statistical inference for temporal sequence of somatic mutations in cancer genomes
Source: BMC Med Genomics. 2018 Apr 20;11(Suppl 2):29. doi: 10.1186/s12920-018-0352-z (PMC5918460; doi:10.1186/s12920-018-0352-z)
Supplement: Supplementary file 2 — Figure S1. Determination of the minimum sample size for the experiments (a) 5 percentile of the 100,000 random re-sampling experiments with the number of cases (b) Distribution of the frequency for the mutant gene pairs. (PDF 23 kb) [file 12920_2018_352_MOESM2_ESM.pdf]

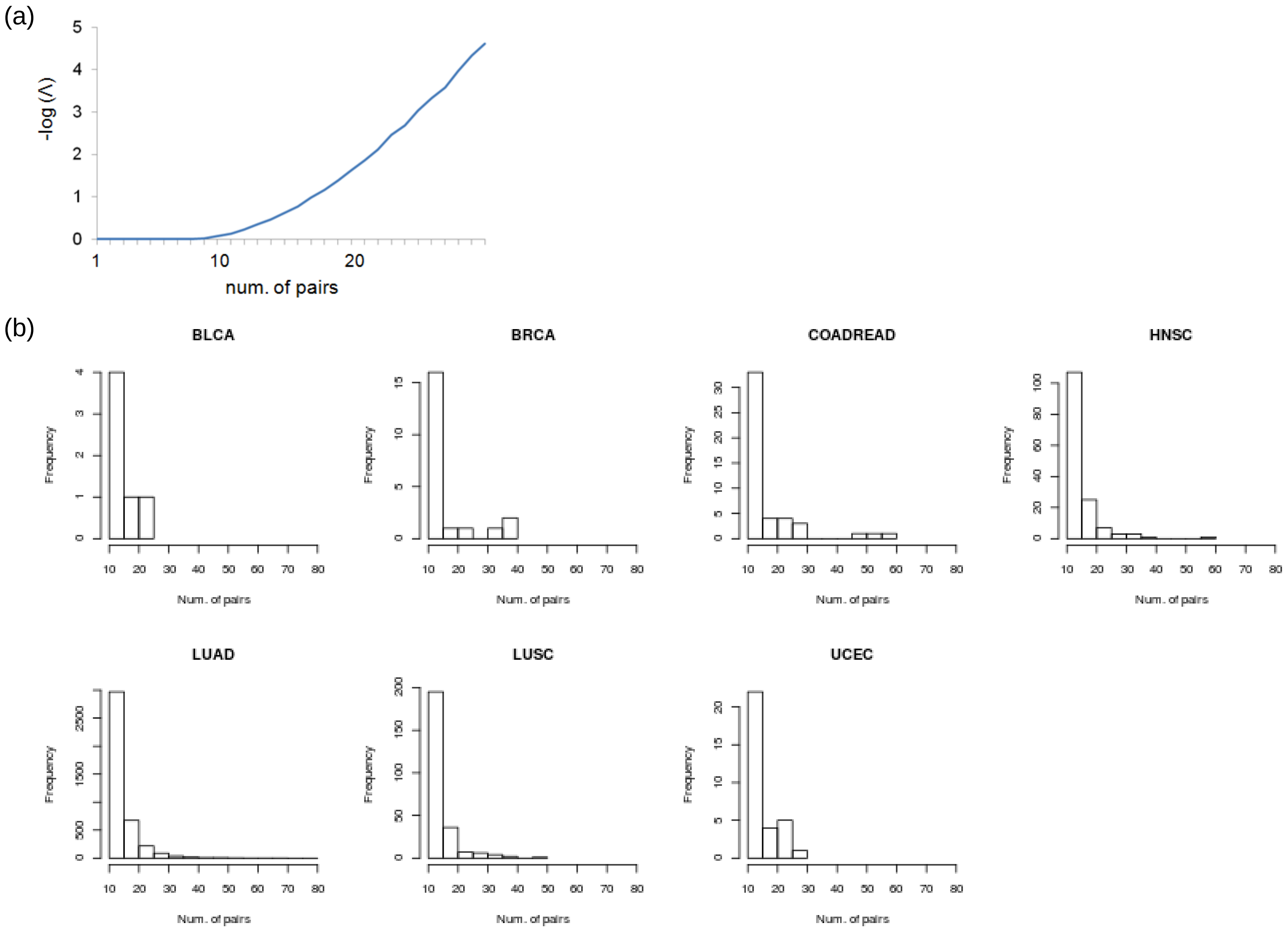

Supplementary Figure S1. Determination of the minimum sample size for the experiments. (a) 5 percentile of the 100,000 random re-sampling experiments with the number of cases (b) Distribution of the frequency for the mutant gene pairs
