# Supplementary material for: Population-based statistical inference for temporal sequence of somatic mutations in cancer genomes
Source: BMC Med Genomics. 2018 Apr 20;11(Suppl 2):29. doi: 10.1186/s12920-018-0352-z (PMC5918460; doi:10.1186/s12920-018-0352-z)
Supplement: Supplementary file 9 — Figure S2. Hotspot mutation and CCF. (a) Maximum value of CCF in COADREAD. Each dot means a tumor patient with a somatic mutation in APC hotspots. (b) Distribution of CCFs for APC and TP53 mutations in COADREAD. The figure is plotted for the patients with APC:Q1387 hotspot mutation. Red is CCF distribution for APC and blue is for TP53. (PDF 35 kb) [file 12920_2018_352_MOESM9_ESM.pdf]

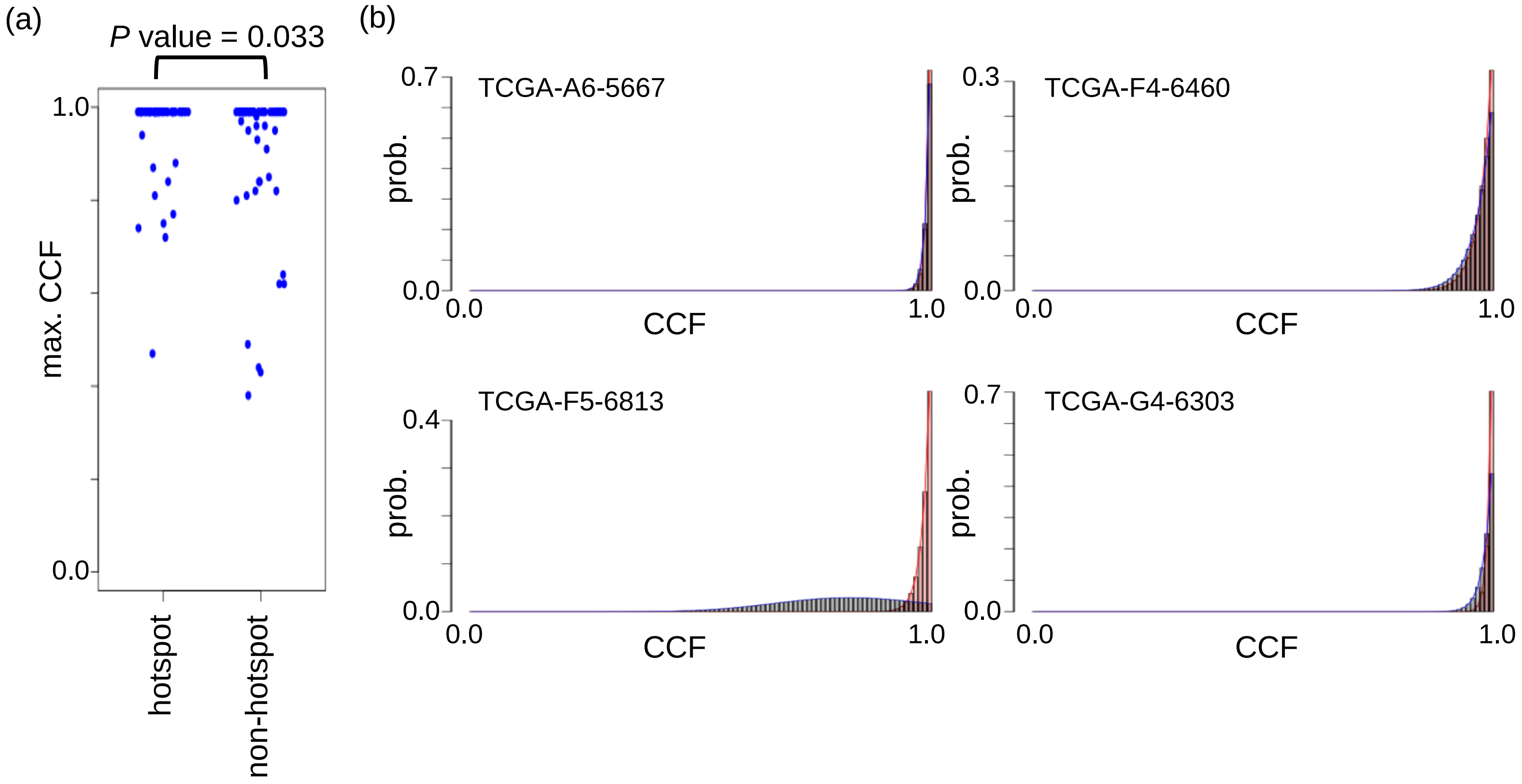

Supplementary Figure S2. Hotspot mutation and CCF. (a) Maximum value of CCF in COADREAD. Each dot means a tumor patient with a somatic mutation in APC hotspots. (b) Distribution of CCFs for *APC* and *TP53* mutations in COADREAD. The figure is plotted for the patients with *APC:Q1387* hotspot mutation. Red is CCF distribution for *APC* and blue is for *TP53*.
